# Biochemical and Molecular Mechanisms of Plant-Microbe-Metal Interactions: Relevance for Phytoremediation

**DOI:** 10.3389/fpls.2016.00918

**Published:** 2016-06-23

**Authors:** Ying Ma, Rui S. Oliveira, Helena Freitas, Chang Zhang

**Affiliations:** ^1^Centre for Functional Ecology, Department of Life Sciences, University of CoimbraCoimbra, Portugal; ^2^Department of Environmental Health, Research Centre on Health and Environment, School of Allied Health Sciences, Polytechnic Institute of PortoVila Nova de Gaia, Portugal; ^3^Centro de Biotecnologia e Química Fina, Escola Superior de Biotecnologia, Universidade Católica PortuguesaPorto, Portugal; ^4^Chuzhou UniversityChuzhou, China

**Keywords:** plant growth promoting microorganisms, root exudates, heavy metals, molecular bases, phytoremediation

## Abstract

Plants and microbes coexist or compete for survival and their cohesive interactions play a vital role in adapting to metalliferous environments, and can thus be explored to improve microbe-assisted phytoremediation. Plant root exudates are useful nutrient and energy sources for soil microorganisms, with whom they establish intricate communication systems. Some beneficial bacteria and fungi, acting as plant growth promoting microorganisms (PGPMs), may alleviate metal phytotoxicity and stimulate plant growth indirectly via the induction of defense mechanisms against phytopathogens, and/or directly through the solubilization of mineral nutrients (nitrogen, phosphate, potassium, iron, etc.), production of plant growth promoting substances (e.g., phytohormones), and secretion of specific enzymes (e.g., 1-aminocyclopropane-1-carboxylate deaminase). PGPM can also change metal bioavailability in soil through various mechanisms such as acidification, precipitation, chelation, complexation, and redox reactions. This review presents the recent advances and applications made hitherto in understanding the biochemical and molecular mechanisms of plant–microbe interactions and their role in the major processes involved in phytoremediation, such as heavy metal detoxification, mobilization, immobilization, transformation, transport, and distribution.

## Introduction

Soil contaminated with heavy metals has become a serious worldwide problem due to geologic and anthropogenic activities. Heavy metals are non-degradable and thus persist indefinitely in the environment. As an alternative to physical and chemical methods, the use of hyperaccumulating plants and beneficial microbes has been a promising approach to clean up metal contaminated soils through extraction (phytoextraction), stabilization (phytostabilization), and/or transformation (phytovolatilization) process (Lebeau et al., 2008; Glick, 2010).

**FIGURE 1:**
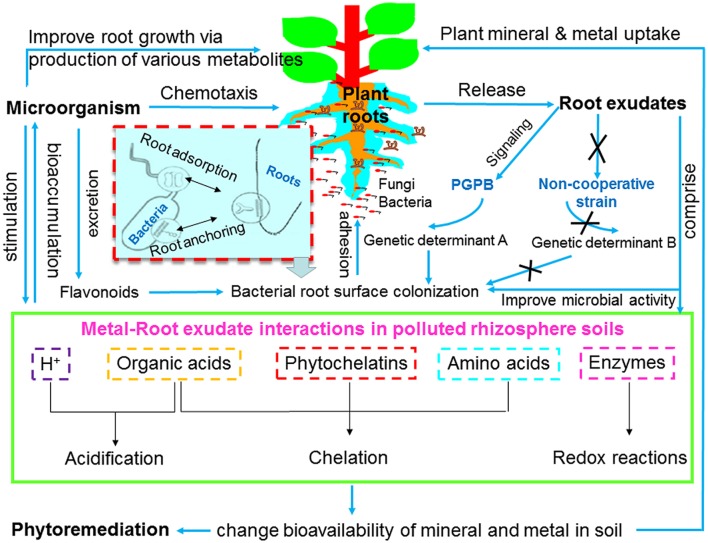
**Schematic overview of mechanisms of plant-microbe-metal interactions.** PGPB, plant growth promoting bacteria.

Root exudates and microorganisms are important components of rhizosphere ecology and play important roles in changing the bioavailability of metals and nutrients. Root exudates provide microbes an abundant source of energy and nutrients for microbes, and in return, microbes stimulate exudation from plant roots. In the co-evolutionary process, plants and their associated microbes coexist or compete for survival in the changing environment, and their relationships, either beneficial or detrimental are of significant importance for both partners. Root exudates are known to enhance mobility of metals and nutrients by (i) acidification due to proton (H^+^) release or by forming organic/amino acid-metal/mineral complexes; (ii) intracellular binding compounds (e.g., phytochelatins, organic acids, and amino acids); (iii) electron transfer by enzymes in the rhizosphere (e.g., redox reactions); and (iv) indirectly stimulating rhizosphere microbial activity (e.g., survival, growth, propagation, and functioning), therefore enhancing phytoremediation efficiency (Ström et al., 2002; Pérez-Esteban et al., 2013; Sessitsch et al., 2013; **Figure 1**).

**FIGURE 2:**
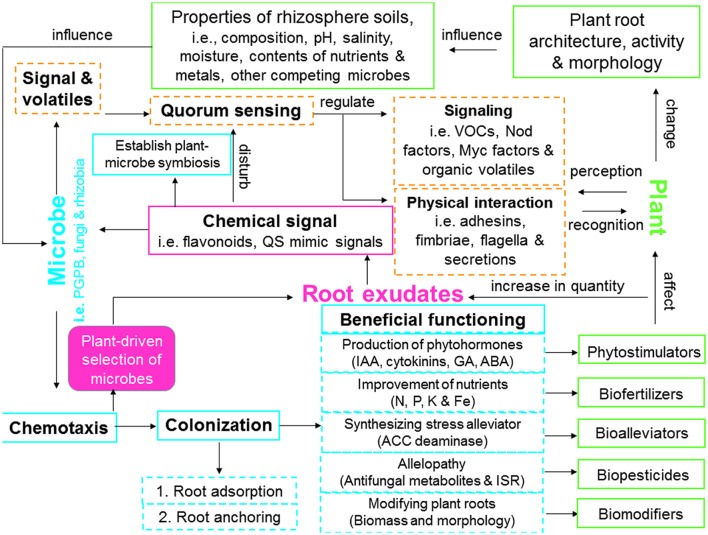
**Model showing plant–microbe interactions including signaling and communication, establishment and functioning of associative symbiosis that can be involved in enhancing phytoremediation efficiency.** QS, quorum sensing; ABA, abscisic acid; ACC, 1-aminocyclopropane-1-carboxylate; GA, gibberellic acid; IAA, indole-3-acetic acid; ISR, induced systemic resistance; VOCs, volatile organic compounds.

Microbes can enhance phytoremediation in different manners: expedite plant biomass, increase (phytoextraction) or decrease (phytostabilization) metal availability in soil, as well as facilitate metal translocation from soil to root (bioaccumulation) or from root to shoot tissues (translocation; Ma et al., 2011a, 2013; Rajkumar et al., 2012; **Figure 1**). In metal rich natural (serpentine soil) and anthropogenic contaminated habitats (e.g., mine waste and fly ash), microbes are able to tolerate considerable high concentrations of metals, and to evolve resistance strategies (Kumar and Nagendran, 2009; Ma et al., 2015c). There are several advantages in using plant growth promoting microorganisms (PGPMs) rather than chemical amendments in phytoremediation, because the microbial metabolites produced in the rhizosphere *in situ* are biodegradable and less toxic (Rajkumar et al., 2012). Metal resistant PGPM have been widely investigated for their potential to improve plant growth, alleviate metal toxicity, and immobilize/mobilize/transform metals in soil, which may help to develop new microbe-assisted phytoremediation and restoration strategies. Arbuscular mycorrhizal fungi (AMF), particularly those isolated from metalliferous sites, are capable of boosting plant growth (Orłowska et al., 2013) and nutrient uptake (Guo et al., 2013), reduce metal induced toxicity (Meier et al., 2011), change metal availability through alteration of soil pH (Rajkumar et al., 2012) and affect metal translocation (Hua et al., 2009). During greenhouse studies with sunflower grown in soils contaminated with three different Cd concentrations, successful AMF colonization by *Rhizophagus irregularis* resulted in an enhanced phytoextraction of Cd, whereas *Funneliformis mosseae* enhanced phytostabilization of Cd and Zn (Hassan et al., 2013). These results indicate that the two root associated AMF may adopt several mechanisms to trigger either metal mobilization or immobilization, therefore contributing directly to phytoextraction or phytostabilization processes (Ma et al., 2011a). In addition, plant growth promoting bacteria (PGPB) possessing single or multiple traits such as alleviation of metal toxicity (metal resistant bacteria), alteration of metal availability (metal immobilizing or mobilizing bacteria), production of siderophores [siderophores producing bacteria (SPB)], phytohormones [indole-3-acetic acid (IAA) producing bacteria] and biochelator (organic acid- or biosurfactants-producing bacteria), fixation of nitrogen [nitrogen fixing bacteria (NFB)], and solubilization of mineral nutrients (phosphate or potassium solubilizing bacteria) have been widely proposed as effective bioinoculants for microbe-assisted phytoremediation (Ma et al., 2011a, 2015a,b,c; Rajkumar et al., 2012; Ahemad and Kibret, 2014; **Figure 2**). Ma et al. (2011a) have extensively reviewed the diversity and ecology of metal resistant PGPB and their potential as phytoremediation enhancing agents in metal contaminated soils. Due to the dual role of these microbial inoculants, the inoculation of PGPB can lead to increased plant biomass and enhanced metal mobilization or immobilization in soil.

**FIGURE 3:**
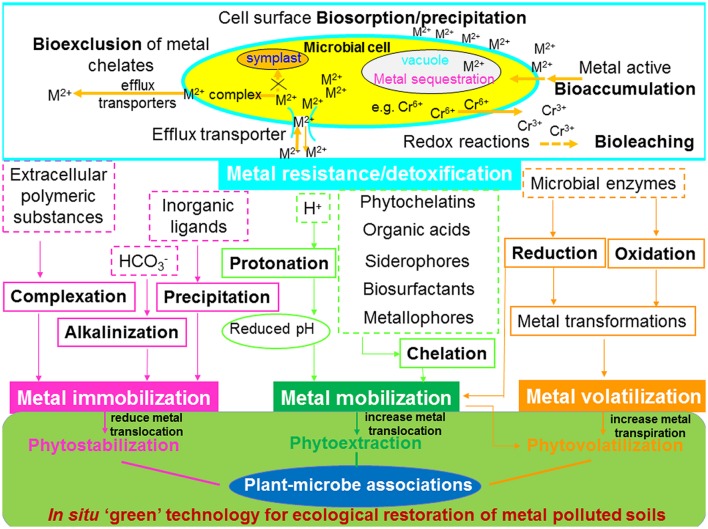
**Effects of functioning of plant-microbial associations on the biogeochemical cycling of heavy metals and their implication for phytoremediation.** M^2+^, divalent metal ion.

The interactions between root exudates and microorganisms in the rhizosphere have been recognized as a critical component of plant growth in phytoremediation (Badri et al., 2009; Segura et al., 2009). However, the mechanisms underlying plant-microbe-metal interactions remain elusive. This article attempts to review the recent advances and applications toward understanding the biochemical and molecular mechanisms of plant–microbe interactions and their role in metal phytoremediation processes. In the following sections, we will elaborate on the mechanisms underlying plant-microbe-metal interactions in the rhizosphere, namely: (1) plant–microbe interactions (molecular signaling and perception, quorum sensing (QS), and establishment of associative symbiosis; **Figure 2**); and (2) heavy metals versus plant–microbe interactions (role of plant-microbe-metal interactions in metal detoxification, mobilization, immobilization, transformation, transport, and distribution; **Figure 3**).

## Plant–Microbe Interactions

Root exudates are defined as organic chemicals released by living and intact root system in certain stages of plant growth. The components of root exudates and their rhizosphere functions are summarized in **Table 1**. Root exudates play an important role in phytoremediation, as they induce the capability of host plants to actively adapt to and survive under metal stress conditions by either allelopathic functions (affecting the growth of rhizosphere microbes and other plants), or detoxification process (including adsorption, chelation, transformation, and inactivation of metals). In addition, root exudates, particularly organic acids are able to bind metal ions, therefore influencing metal mobility, solubility and bioavailability in soil (Chiang et al., 2011; Luo et al., 2014; **Figures 1** and **3**). Kim et al. (2010) reported that citric acid and oxalic acid from *Echinochloa crusgalli* significantly enhanced both translocation and bioaccumulation of metals (Cd, Cu, and Pb), suggesting that organic acids can be considered natural chelating agents to enhance phytoextraction. However, some components of root exudates do not influence metal availability or have a negative impact on metal mobilization (Zhao et al., 2001). The low molecular weight organic acids (LMWOAs), such as oxalate, released by both ectomycorrhizal and non-mycorrhizal seedlings of Scots pine contributed to metal immobilization through formation of stable metal complexes in soil (Johansson et al., 2008).

**Table 1:** Components of root exudates and their roles in the rhizosphere.

Classification	Species	Rhizosphere function	Reference
Organic acids	Acetic, aconitic, adipic, butyric, citric, cyclic, formic, fumaric, gluconic, glutaric, glycolic, glyoxylic, hydroxybutyric, indole-3-acetic, isocitric, lactic, maleic, malic, malonic, oxalic, piscidic, propionic, pyruvic, succinic, tartaric, valeric	Nutrient and energy sources, chemoattractant signals to microbes, chelators/adsorbents of insoluble mineral nutrients, acidifiers of soil, *nod* gene inducers, antibacterial agents	Mucha et al., 2005; Magdziak et al., 2011; Ramachandran et al., 2011; Vranova et al., 2013

Amino acids	α-alanine, β-alanine, γ-aminobutyric acid, arginine, asparagine, aspartic acid, cysteine, cystine, glutamic acid, glutamine, glycine, histidine, homoserine, isoleucine, leucine, lysine, methionine, phenylalanine, proline, serine, threonine, tryptophan	Nutrient and energy sources, chelators of insoluble mineral nutrients, chemoattractant signals to microbes	Yao et al., 2005; Ma et al., 2009a,b, 2011a; Ahemad and Kibret, 2014; Glick, 2014

Saccharides	Arabinose, fructose, fucose, galactose, glucose, lactose, mannose, raffinose, rhamnose, ribose, sucrose, xylose	Nutrient and energy sources, anchoring of bacteria to plant surfaces	Guibaud et al., 2005; Juwarkar et al., 2007; Sheng et al., 2008; Slaveykova et al., 2010; Venkatesh and Vedaraman, 2012

Phenols	Caffeic acid, ferulic acid, flavonoids/bioflavonoids, *N*-hexanoyl-D,L-homoserine-lactone, 7-hydroxy-6-methoxy coumarin, isoflavonoids, neoflavonoids, pyrocatechol, quercetin, strigolactones, styrene	Nutrient and energy sources, chemoattractant signals to microbes, chelators of insoluble mineral nutrients, microbial growth promoters, *nod* gene inducers or inhibitors in rhizobia, inductors of resistance against phytopathogens	Dakora and Phillips, 2002; Zhao et al., 2005; Steinkellner and Mammerler, 2007; Steinkellner et al., 2007; von Rad et al., 2008; Hofmann, 2013

Enzymes	Amylase, DNase, phosphatase, polygalacturonase, protease, RNase, sucrase, urease, xylanase	Release of phosphorus from organic molecules, transformations of organic matter in soil	Loh et al., 2002; Gonzales-Chavez et al., 2004; Ahemad and Kibret, 2014; Wu et al., 2014

Vitamins	*p*-aminobenzoic acid, ascorbic acid, biotin, β-carotene, folic acid, niacin, pantothenate, pyridoxine, riboflavin, thiamin, thioctic acid, tocopherol, vitamin B12	Stimulation of plant and microbial growth, nutrient source, resistance to soil pathogens, facilitation of organic pollutant degradation, induction of plant–microbe symbioses	Kafkewitz et al., 1996; Bertin et al., 2003; Vranova et al., 2013

Others	Bilineurine, bradyoxetin, glomalin, inositol, nicotinic acid, rhamnolipids, somatropin, surfactants	Stimulation of plant and microbial growth, regulators of symbiotic expression of nodulation genes (*nod*, *nol*, *noe*)	Loh et al., 2002; Vijayan et al., 2013

In general, plants possess the ability to select their own root microflora from the surrounding soil and each particular plant species has a characteristic group of associated microbes (Hartmann et al., 2009). This process is most likely to be linked directly to the quantity and composition of root exudates as well as properties of rhizosphere soil. Based on coevolutionary pressures, interactions between plants and their associated microbes are highly dynamic in nature (Chaparro et al., 2013; **Figure 2**). In the rhizosphere, plants can effectively communicate with their neighboring soil microorganisms by exuding chemicals or signals (signaling molecules and their perception, QS), while their associated microbes may establish an efficient associative symbiosis with plants by triggering host functional signals (e.g., microbial chemotaxis and colonization; Doornbos et al., 2012; Drogue et al., 2012; Bulgarelli et al., 2013; **Figure 2**).

### Signaling Molecules and Their Perception

Extensive communication between plants and microbes via various signaling molecules plays a significant role in maintaining the growth of both partners. These include plant-released chemical signals that are recognized by the microbes and microbial signals and volatiles that trigger changes in plant physiology (**Figure 2**).

#### Plant-Released Signals

Root exuded flavonoids are known as the key signaling components in a number of plant–microbe interactions (e.g., mycorrhiza formation, establishment of legume-rhizobia symbiosis; Steinkellner et al., 2007; **Figure 2**). Flavonoids play a significant role in AMF spore germination, hyphal growth, differentiation, and root colonization in AMF-plant interactions (Badri et al., 2009; Mandal et al., 2010). At the initial stage of AMF-plant association, flavonoids exhibit an AMF fungal genus- and even species specific effect on the pre-symbiotic hyphal growth (Scervino et al., 2005b). By linking alterations of flavonoid pattern in mycorrhizal roots to the developmental stage of AMF symbiosis, Larose et al. (2002) observed intermediate levels of flavonoids in roots during root penetration and the early establishment of AMF, whereas, high levels of flavonoids (such as phytoalexin and medicarpin) at a later stage of root colonization. Once plants are well-colonized by AMF, the flavonoid pattern is dramatically changed and this change plays a regulatory role in plant-AMF interaction (Badri et al., 2009). Moreover, the stimulatory effects of flavonoids on AMF growth might be compromised, because each flavonoid can also exert a negative or neutral effect on different fungi due to its specificity involved in mycorrhizal symbiosis formation (Scervino et al., 2005a). So far, during pre-symbiotic growth, the role of flavonoids and other phenolic acids in AMF association is still unclear.

Apart from their function in the AMF-host symbiosis, flavonoids are able to promote the growth of host-specific rhizobia by serving as chemoattractants and inducers of nodulation (*nod*) genes involved in the synthesis of lipochitin–oligosaccharide signaling molecules, the Nod factors (Perret et al., 2000; Mandal et al., 2010). The flavonoids released by plant roots are recognized by rhizobial *nodD* proteins, transcriptional regulators that bind directly to a signaling molecule and are able to synthesize and export *nod* genes. Upon exposure to Nod factors, the root hair cells infection and nodule formation in the host are stimulated. Therefore, specific flavonoids induce not only *nod* gene expression, but also rhizobial chemotaxis and bacterial growth (Bais et al., 2006). This specificity enables rhizobia to recognize their correct host plants and then attach to the root hairs. In addition, some other flavonoid related compounds, such as isoflavonoids (e.g., daidzein and genistein) and plant flavone (e.g., luteolin) can also effectively induce rhizobial *nod* gene expression (Juan et al., 2007). To our knowledge, the data on the implication of flavonoid as signaling compounds in other plant–microbe interactions are relatively limited.

#### Microbial Signals

Free-living microbes (e.g., PGPB, fungi, and rhizobia) are able to alter the chemical composition of root exudates and thus plant physiology via releasing of various signaling molecules, such as volatile organic compounds (VOCs), Nod factors, Myc factors, microbe-associated molecular patterns (MAMPs) and exopolysaccharides (Goh et al., 2013; **Figure 2**). Bacterial VOCs (such as acetoin and 2,3-butanediol) can establish the communication with plants, trigger plant defense and growth promotion mechanisms by enabling host plants to colonize nutrient (e.g., sulfur and iron) poor soils (Bailly and Weisskopf, 2012), which are common in phytoremediation scenarios. Hofmann (2013) recently demonstrated that VOCs released by *Bacillus* B55 significantly contributed to *Nicotiana attenuata* sulfur nutrition. Ortíz-Castro et al. (2009) have extensively reviewed the occurrence, function and biosynthesis of bacterial volatiles and their role in positive plant–microbe interactions. The available information indicates that VOC emission has a crucial impact in most PGPMs of PGPB by acting as bioprotectants [via induced systemic resistance (ISR); Ryu et al., 2004], biopesticides (via antibiotic functions; Trivedi and Pandey, 2008) and phytostimulators (via triggering hormonal signaling networks; Zhang et al., 2008; **Figure 2**). These functions can contribute to improve plant growth, which is fundamental for successful phytoremediation. Ryu et al. (2004) reported that VOCs secreted by *Bacillus subtilis* and *B. amyloliquefaciens* can activate ISR in *Arabidopsis* seedlings challenged with the soft-rot pathogen *Erwinia carotovora* subsp. *carotovora*. Moreover, some bioactive VOCs produced by PGPB such as ammonia, butyrolactones, hydrogen cyanide, phenazine-1-carboxylic acid, alcohols, etc. are able to affect mycelium growth and sporulation of different fungal species (Kai et al., 2009). In this regard, VOCs can be used for communication between bacteria and their eukaryotic neighbors. Furthermore, signaling molecules synthesized by AMF (Myc factor) and rhizobia (Nod factors) are able to modulate root system architecture (such as stimulation of lateral root branching, formation of new organs and nodule), therefore facilitating symbiotic infections or nodule organogenesis in the course of evolution (Olah et al., 2005; Maillet et al., 2011). The Nod factor signaling pathway can be also affected by the Myc factor, leading to AMF formation (Maillet et al., 2011). In addition, plants have evolved a large set of defense mechanisms after pathogen perception via the plant innate immune systems pattern recognition receptors. Phytopathogen recognition can be achieved through MAMPs, which are known as biotic elicitors of non-specific immunity in plants (Newman et al., 2013). Varnier et al. (2009) found that novel MAMPs (rhamnolipids) released from *Pseudomonas aeruginosa* protected grapevine against pathogenic fungi. Recently, MAMPs from three PGPB (*Stenotrophomonas maltophilia*, *Chryseobacterium balustinum*, and *Pseudomonas fluorescens*) were able to trigger germination and metabolism of *Papaver somniferum* (Bonilla et al., 2014).

### Quorum Sensing

Quorum sensing is a bacterial cell–cell communication process, whereby a coordinated population response (such as monitoring of population density, collective alteration of bacterial gene expression) is controlled by diffusible signaling molecules produced by individual bacterial cells (Daniels et al., 2004; **Figure 2**). The QS induced processes such as sporulation, competence, antibiotic and biofilm production, have been widely documented in plant–microbe interactions (Williams and Camara, 2009). QS signals, such as *N*-acyl-L-homoserine lactones (AHLs) are the essential components of this communication system. AHL quorum signals can enhance or inhibit diverse phenotypes depending on the bacteria being beneficial or pathogenic (Ortíz-Castro et al., 2009). AHLs are commonly found in many Gram-negative pathogenic bacteria (e.g., *P. aeruginosa*, *Rhizobium radiobacter*, and *Erwinia carotovora*) and/or PGPB (e.g., *Burkholderia graminis* and *Gluconacetobacter diazotrophicus*; Cha et al., 1998), which can be used to control a broad range of bacterial traits (such as symbiosis, virulence, competence, conjugation, motility, sporulation, biofilm, and antibiotic production; Fuqua et al., 2001). Conversely, bacterial AHLs can be recognized by plants, thereafter modulating tissue-specific gene expression, plant growth homeostasis, and defense response (Daniels et al., 2004). Recently, Pérez-Montaño et al. (2011) reported that similar pattern of AHLs (e.g., *N*-octanoyl homoserine lactone and its 3-oxo and/or 3-hydroxy derivatives) released by rhizobia *Sinorhizobium fredii*, *Rhizobium etli*, and *R. sullae* were involved in interactions with their host legumes. Similarly, von Rad et al. (2008) demonstrated that the contact of *Arabidopsis thaliana* roots with the bacterial QS molecule *N*-hexanoyl-homoserine lactone (C6-HSL) caused distinct transcriptional changes in legume tissues. Moreover, the AHL mimic compounds (e.g., furanones signals) secreted by higher plants (such as soybean, rice, and barrel clover) and other eukaryotic hosts can disrupt or manipulate QS-regulated behaviors among bacterial population (Pérez-Montaño et al., 2013). The AHL mimics can antagonize AHL-type behaviors by binding to the AHL receptor (e.g., LuxR) due to their structural similarities to bacterial AHLs, therefore affecting bacterial AHL-signaling (Bauer and Mathesius, 2004). Plants may adopt AHL mimics to communicate with specific bacteria to protect them from pathogens. In addition, root exudates (e.g., flavonoid and genistein) play an important role in bacterial QS communication, since they can chemotactically attract rhizobia toward, adhere to and colonize legume roots, as well as regulate expression of rhizobial nodulation genes [such as *nod* and rhizosphere-expressed (*rhi*) genes] in plant tissues (Loh et al., 2002). Strikingly, QS can be prevented (so called quorum quenching) by bacterial VOCs that can significantly affect the AHL output by the producer strain, neighboring pathogenic and/or beneficial rhizobacteria (Dong et al., 2001; Chernin et al., 2011).

The information discussed above suggests that plants and bacteria have acquired mechanisms to sense and respond to each other’s signaling molecules. Further research is needed to select the key plant and microbial signaling molecules and study their functions of mediating this interkingdom communication in the rhizosphere, which could develop novel strategies to enhance phytoremediation.

### Establishment of Associative Symbiosis

#### Microbial Chemotaxis

Root exudates are composed by diverse compounds (**Table 1**), which act as chemoattractant signals and/or sources of carbon and nitrogen for microbes (Bais et al., 2006; Compant et al., 2010), therefore creating a unique environment in the rhizosphere. However, the rhizomicrobiome composition differs according to root exudate composition, as it changes along the root system due to plant genotypes and development stages (Chaparro et al., 2013). Consequently, the microbial chemotaxis toward particular root-exuded compounds is an important trait for plant-driven selection of microbes and their colonization (Doornbos et al., 2012; **Figure 2**). It has been established that plants can elicit crosstalk to microorganisms by secreting root exudates as signaling molecules, enabling colonization by some beneficial bacteria while inhibiting the other pathogenic bacteria (Rudrappa et al., 2008; Compant et al., 2011). Rudrappa et al. (2008) demonstrated that malic acid secreted from roots of *Arabidopsis thaliana* recruits the rhizobacterium *B. subtilis* to the root and this interaction plays a significant role in protecting the plant against the foliar pathogen *Pseudomonas syringae*. The demonstration that roots selectively exude organic compounds to effectively signal bacteria and fungi underlines the role of plant metabolites in recruitment of beneficial microbes and in plant–microbe interactions. In addition to chemotaxis, electrotaxis toward electric gradients generated by plant roots is considered as a possible mechanism for initiating rhizobacterial colonization (Lugtenberg and Kamilova, 2009).

#### Microbial Colonization

Root colonization is the competitive process and critical step in establishment of plant–microbe association, which is potentially affected by characteristics of both host plants and their associated microbes (Reinhold-Hurek and Hurek, 2011). In general, the process of microbial colonization in plant rhizosphere or tissue interior includes: migration toward root (root adsorption), surface attachment (root anchoring), distribution along root, as well as survival and population growth (**Figures 1** and **2**). For microbial endophytes, one more step is required that is entry into plant tissue interior and formation of micro-colonies either inter- or intracellularly (Ma et al., 2011b, 2015a; Wang et al., 2016).

Bacteria are able to rapidly and adequately alter their cell surface, thereby effectively attaching and colonizing plant roots. In the colonization process, a significant role is played by cell-surface biopolymers including proteins, glycoproteins, glycopeptidolipids, and other macromolecular metabolites (Kamnev, 2008). Among some of cell-surface protein characterized, such as bacterial major outer membrane protein (MOMP) and lectin, their involvement along with adhesion (flagella-driven chemotaxis toward root exudates), root adsorption and cell aggregation of bacteria in host recognition and root colonization process has been demonstrated (Lugtenberg and Kamilova, 2009; Compant et al., 2010). Furthermore, type IV pili and twitching motility are also involved in plant root colonization process. Böhm et al. (2007) studied the role of the type IV pili in rice colonization process of *Azoarcus* spp. using a deletion mutant of pilA and insertional mutant of pilT that was abolished in twitching motility. The results demonstrated that the retractile force mediated by PilT is unessential for bacterial colonization of plant surface, but the twitching motility is necessary for plant invasion and tissue interior establishment. Moreover, the role of two component system of *Pseudomonas fluorescens* ColR/S in competitive tomato root tip colonization has been reported. ColR/S system regulates methyltransferase/WapQ operon and maintains the outer membrane integrity for efficient colonization (De Weert et al., 2009). The colonization process is important to understand the plant-bacteria interactions and the potential of bacteria to establish themselves in the plant environment as biofertilizers, biocontrol agents, and facilitators of phytoremediation processes.

Microbial colonization can be traced by tagging them with certain molecular marker such as green fluorescent protein or β-glucosidase followed by microscopy (Reinhold-Hurek and Hurek, 2011). Although, understanding of the molecular mechanism involved in microbial colonization process is not well-understood, resemblance of colonization methods between pathogenic bacteria and PGPM have been suggested by Hardoim et al. (2008).

#### Functioning of Associative Symbiosis

Plant–microbial communication is a critical process to characterize the belowground rhizosphere zone, which can be beneficial to host plants and microbes. The metal resistant beneficial microbes (bacteria and AMF) are often used as bioinoculants to affect metabolic functions and membrane permeability of root cells and thus to enhance the establishment, growth and development of remediating plants in metal contaminated soils through: (1) facilitating mineral phytoavailability (N, P, K, Ca, and Fe) by acting as biofertilizers; (2) modulating phytohormones balance by acting as phytostimulators; (3) reducing ethylene synthesis by acting as stress bioalleviators; (4) preventing deleterious effects of phytopathogens via production of antifungals and ISR, by acting as biopesticides; and (5) modifying root biomass and morphology by acting as biomodifiers (Lugtenberg and Kamilova, 2009; Glick, 2010; Ma et al., 2011a; Miransari, 2011; Rajkumar et al., 2012; Ahemad and Kibret, 2014; **Figure 2**).

Currently, a numbers of studies have manifested that some beneficial microbes can help plants acquire sufficient mineral nutrients (such as N, Ca, Fe, Mg, and P) in metal contaminated soils, therefore develop longer and prosperous root system and get better established during the early growth stage, which is highly desirable in heavy metal phytoremediation (Ahemad and Kibret, 2014; **Figure 2**). Examples are NFB (such as rhizobia, rhizobacteria, and endophytic bacteria) and nutrient-absorbing AMF, which can improve the fertility of polluted soils for plant growth by catalyzing the reduction of atmospheric N_2_ to biologically available ammonium (Navarro-Noya et al., 2012). Wani et al. (2007) reported that the inoculation of *Vigna radiata* L. with the NFB *Bradyrhizobium* sp. RM8 conferred tolerance to plants grown under metal stress by enhancing N concentration in roots and shoots. Similarly, the AMF *Glomus* spp. benefited plant growth and nutrient (N, P, and K) uptake by leguminous trees grown on Pb/Zn mine tailings. Further, P is one of the major macronutrients required for plant growth, however, most P compounds are not readily soluble in soil and are hence unavailable to plants (Harris and Lottermoser, 2006). The insoluble P compounds in soil can be solubilized by enzymes, organic acids and/or chelating agents excreted by both plants and microbes. One example are P solubilizing microbes (PSMs), which are widely used as inoculants to improve P uptake and plant yield by dissolving various sparingly insoluble P sources with a decrease in the rhizosphere pH (Jeong et al., 2013). Some microorganisms have the ability to couple biologically liberated P with the formation of metal phosphate biominerals, through either the accumulation of high concentrations of P cleaved from glycerol 2-phosphate at microbial cell surface, or the microbial P cycling process (Lloyd and Lovley, 2001). Strikingly, the distribution and activity of phosphate solubilizing bacteria (PSB) and their subsequent effects on P solubilization are determined by exogenous P concentration in soil (Sharma et al., 2013). In the presence of soluble P in soil, the solubilization of insoluble P by some PSB can either be repressed or not affected. However, there are few studies on the number of total PSB and their ability to solubilize P in soil. In addition, microbes can also precipitate highly insoluble metal sulfides, leading to the removal of toxic metals from solutions. This may provide a more attractive option for microorganisms to increase their resistance to metals *per se*. Sharma et al. (2000) reported that *Klebsiella planticola* precipitated cadmium through the formation of sulfide from thiosulfate. Moreover, siderophores and protons can also be specifically produced by soil microorganisms in response to iron (Fe) deficiency in soil. Recently, the role of siderophore producing microbes (SPMs) such as bacteria and fungi in Fe acquisition by different plant species as well as the mechanisms behind their promotion of Fe acquisition have been widely studied (Kajula et al., 2010; Ma et al., 2011c; Gaonkar and Bhosle, 2013). Ma et al. (2011c) demonstrated that the bacterial catechol and hydroxamate siderophores produced by *Psychrobacter* sp. SRS8 enhanced the growth of *Ricinus communis* and *Helianthus annuus* in Ni contaminated soil by a simultaneous enhancement of Fe solubilization and uptake. Bacterial siderophores also play a crucial role in the generation/regulation of hormones in plants under metal stress. Chelation through binding toxic metals to siderophores triggers the enhancement of plant Fe uptake capacity and the decrease of free metal ion concentration, thus leading to the attenuation of hormone synthesis inhibition (Dimkpa et al., 2008). Bacteria, such as *Arthrobacter* sp. and *Leifsonia* sp. (Actinobacteria), *Polaromonas* sp. and *Janthinobacterium* sp. (Betaproteobacteria), were previously reported to accelerate the dissolution and mobilization of mineral nutrients (such as Fe, Mn, and K) for soil fertility (Abdulla, 2009; Uroz et al., 2009).

Plant associated microbes can also produce phytohormones such as IAA, cytokinins, gibberellic acid, abscisic acid and others, even under stress conditions, thereby modulating the hormonal balance in plants and their response to stress (Ma et al., 2011a; Ullah et al., 2015; **Figure 2**). The IAA synthesized by microbes, together with endogenous plant IAA, cannot only stimulate root exudation and proliferation of lateral and adventitious roots, but also induce the synthesis of ACC synthase (Glick, 2010). Recently, Chen B. et al. (2014) found that the IAA producing endophytic bacterium *Sphingomonas* SaMR12 increased IAA concentration in plant tissues and the growth of *Sedum alfredii*. Similarly, Ma et al. (2013) assessed the potential of *Phyllobacterium myrsinacearum* RC6b to produce IAA in culture media containing various concentrations of L-tryptophan. The results indicated that although L-tryptophan is a precursor for bacterial growth and IAA production, high concentrations of L-tryptophan (3, 4, and 5 mg mL^-1^) exert negative effects on bacterial IAA production. The IAA synthesized by RC6b induced significantly greater root growth of *Sedum plumbizincicola* than that of non-inoculated control plants. Furthermore, AMF colonization also has positive effects on plant cell growth and division as a result of fungal hormones production. Yao et al. (2005) demonstrated that inoculation of the AMF *Glomus intraradices* and *Gigaspora margarita* onto seedlings of *Litchi chinensis* significantly increased the IAA and isopentenyl adenosines concentrations in shoots and roots. The changes in endogenous phytohormones level were responsible for morphological alteration induced by AMF inoculation.

Besides the above described plant beneficial mechanisms, under stress conditions soil microorganisms can also enhance plant growth through the synthesis of ethylene production inhibitors [e.g., 1-aminocyclopropane-1-carboxylate (ACC) and rhizobitoxine; Vijayan et al., 2013; Glick, 2014], antimicrobial enzymes (e.g., chitinases, phytoalexins, β-1,3-glucanase, callose, phenolics, and lysozyme; González-Teuber et al., 2010; Saima et al., 2013) and polysaccharides [e.g., exopolysaccharides and extracellular polymeric substances (EPSs); Ashraf et al., 2004; Naseem and Bano, 2014], thus enabling plants to mitigate the negative impact of both biotic (fungi or harmful insects) and abiotic stresses (such as flooding, drought, salinity, and heavy metals; **Figure 2**). One of the key traits related with plant growth promotion is the production of ACC deaminase by PGPB that hydrolyses the plant ethylene precursor ACC into ammonia and α-ketobutyrate (Glick et al., 2007; Glick, 2014). The inoculation of seven Pb-resistant and ACC deaminase-producing endophytic bacterial isolates onto *Brassica napus* grown in metal contaminated sands was found to increase the dry weights of shoots (ranging from 39 to 71%) and roots (from 35 to 123%), compared to the non-inoculated control (Zhang et al., 2011). ACC deaminase-producing microorganisms are able to dilute the negative impact of metal-induced ethylene production in plants, avoiding plant growth inhibition or even death (Glick et al., 2007). Rhizobitoxine is another inhibitor of ethylene synthesis, which cannot only minimize the negative effects of stress-induced ethylene production on nodulation, but also induce foliar chlorosis in soybeans (Vijayan et al., 2013). Prasanna et al. (2013) found that a novel strain *Brevibacillus laterosporus* produced two extracellular chitinase (89.6-kDa four domain and 69.4-kDa two domain) that contribute to its antifungal and insecticidal activities. In addition, some exopolysaccharides-producing plant growth promoting rhizobacteria (PGPR) such as *Proteus penneri*, *P. aeruginosa*, and *Alcaligenes faecalis* were proved to alleviate water stress and improve plant biomass under drought stress (Naseem and Bano, 2014).

Notwithstanding the above, inoculation of efficient fungi and bacteria in compatible host-microorganism-site combination can significantly contribute to modify root morphology and improve plant biomass, which could be a main support for a successful biotechnological application in phytoremediation. AMF play an important role in improving plant establishment, owing to their role in enhancing nutrient uptake and the longer term development of plant root system (Jankong and Visoottiviseth, 2008; Jiang et al., 2016). For example, some species of the genera *Achromobacter*, *Azospirillum*, *Burkholderia*, *Methylobacterium*, and *Psychrobacter* can increase some root morphological traits (Madhaiyan et al., 2007; Molina-Favero et al., 2008; Ma et al., 2009a,b).

In general, plant associated microorganisms are able to promote plant establishment, growth and development by resorting to any one or more of the above mentioned mechanisms. Therefore, those PGPM can be applied not only in agricultural soils for food production, but also in stressful environments for phytoremediation purposes. The effectiveness of PGPM for promoting plant growth depends on the intimate interaction with their host plant and soil characteristics besides their inherent capabilities (Gamalero et al., 2010; Nadeem et al., 2014).

Plant growth promoting microorganisms display various plant-beneficial features, suggesting that the accumulation of the corresponding genes could have been selected in these microbes. The accumulation of the genes contributing to plant beneficial functions might be an intrinsic feature of PGPM. Future studies should focus on discovering preferential associations occurring between certain genes contributing to phytobeneficial traits, which could provide new insights into plant-PGPM interactions.

## Role of Plant-Microbe-Metal Interactions in Phytoremediation

The discovery of plant-microbe-metal interactions sustains the importance of plant–microbe interactions in the biogeochemical cycling of metals and in their application in phytoremediation. It is essential to consider the appropriate combination of plants and microbes involved in applied processes for enhanced phytoremediation efficiency, in order to obtain the best performance from the existing microbe-based technologies. The plant-microbe-metal interactions spanning from both macropartner (higher plants) and micropartner (microorganism) to heavy metals is nevertheless a crucial step in understanding plant metal uptake during geo-bio-interactions (**Figure 3**).

Advances in understanding plant–microbe interactions on metal tolerance and detoxification, together with their functioning on the biogeochemical cycling of heavy metals including metal mobilization/immobilization, translocation and transformation, have led to the development of improved metal bioremediation processes (Bruins et al., 2000; Ma et al., 2011a). The influence of bacterial and fungal activity on metal mobilization or/and immobilization and its use for bioremediation has been reviewed by several researchers (Khan, 2005; Ma et al., 2011a; Rajkumar et al., 2012; Sessitsch et al., 2013; Ahemad and Kibret, 2014). The activities of PGPM, such as metal bioaccumulation, bioleaching and bioexclusion are involved in causing adaptation/resistance/tolerance of microbial communities to heavy metal rich environments. In general, processes such as acidification, chelation and protonation lead to mobilization of metals, whereas precipitation, alkalinization, and complexation cause metal immobilization. However, chemical transformation can cause metal mobilization and/or immobilization. A schematic illustration of the effects of plant–microbial association on the biogeochemical cycling of heavy metals and its implications for phytoremediation is presented in **Figure 3**.

### Metal Detoxification

Heavy metal tolerance/resistance in plants and microbes is a vital prerequisite for plant metal accumulation and microbe-assisted phytoremediation. When plants are subjected to high level of metal contaminants, the stress triggers the plants’ inter-linked physiological and molecular mechanisms in adapting to stressful environment. Mechanisms involved in plant metal tolerance include plant cell wall binding, active transport of ions into cell vacuoles, intracellular complexation with peptide ligands such [as phytochelatins (PCs) and metallothioneins (MTs)], as well as sequestration of metal-siderophore complexes in root apoplasm or soils (Miransari, 2011; **Figure 3**). Among the tolerance mechanisms employed by metal-accumulating plants, the exudation of various compounds, especially LMWOAs is able to stimulate microbial growth, solubilize insoluble or sparingly soluble mineral nutrients (e.g., P, Fe, and Zn), and detoxify some metals (e.g., As, Cd, and Pb; Tu et al., 2004; Li et al., 2013). Root exudation of LMWOAs has been considered one of the most important strategies developed by plants to tolerate high metal concentrations, due to their ability to exclude metals and metalloids (e.g., As, Cr, Cd, and Pb) through chelation in the rhizosphere or apoplast, thereby preventing the metal ions from entering the cell symplast (Magdziak et al., 2011). Some studies found that the diverse LMWOAs, such as citric, oxalic, malic, and succinic acid exuded by agricultural plants under metal stress play a significant role in alleviating metal phytotoxicity (Evangelou et al., 2006; Yuan et al., 2007; Meier et al., 2012).

Moreover, plant associated AMF and bacteria are capable of inducing metal translocation and distribution, thus metal allocation to the inner root parenchyma cells (Kaldorf et al., 1999). The mechanisms involved in microbial metal resistance are summarized schematically in **Figure 3**: (1) cell surface biosorption/precipitation of metals; (2) active eﬄux pumping of metals out of the cell via transporter system; (3) sequestration of metals in intracellular compartments (mainly cell vacuole); (4) exclusion of metal chelates into the extracellular space; and (5) enzymatic redox reaction through conversion of metal ion into a non-toxic or less toxic state.

#### Bioaccumulation

Plant associated microorganisms have been documented to contribute to plant metal resistance through bioaccumulation mechanisms involving interaction and concentration of toxic metals in the biomass of either non-living (biosorption) or living (bioaccumulation) cells (Ma et al., 2011a; Rajkumar et al., 2012; **Figure 3**). Bioaccumulation is a process of intracellular accumulation of metals. It comprises two stages: metabolism-independent passive biosorption (e.g., physical and chemical adsorption, metal ion exchange, chelation, coordination, surface complexation, and microprecipitation), and metabolism-dependent active bioaccumulation (e.g., transport of metal ions into microbial cells including complex permeation, carrier mediated ion pumps and endocytosis; Chojnacka, 2010). Although, the majority of original research has recently focused on biosorption concerning the binding metals by waste materials, the renewable biosorbents (living or dead cells of bacteria, fungi, and plant etc.) have proven to be efficient and economical for the removal of toxic metals from both polluted aqueous solutions and soils (Alluri et al., 2007; Ma et al., 2013). Recently, Ma et al. (2015b) observed that the metal resistant *Bacillus* sp. SC2b was capable of adsorbing significant amounts of metals (e.g., Cd, Pb, and Zn) and bacterial inoculation ameliorated metal toxicity through biosorption, thus exhibiting a protective effect on host plant growth. Dissimilarly, Zafar et al. (2007) studied biosorption of Cr and Cd by metal resistant filamentous fungi *Aspergillus* and *Rhizopus* and found no direct relationship between metal tolerance and biosorption properties of these fungi. The bioaccumulation process is more complex than biosorption and it requires metabolic activity of living cells involving intracellular sequestration (MTs and PCs binding), extracellular precipitation, metal accumulation and complex formation (Gadd, 2004). This process is highly determined by the operational conditions, especially by the presence of metals in the growth medium, as high metal concentrations can inhibit bacterial growth and their bioaccumulation capacity (Chojnacka, 2010). Metal bioaccumulation by various microbes has been widely described in the literature and it was demonstrated that bioaccumulation mechanism can be accounted for both reduced plant metal toxicity and uptake (Ma et al., 2011a; Deng and Wang, 2012; Mishra and Malik, 2013). Velásquez and Dussan (2009) carried out studies on metals (Co, Hg, Fe, and As) biosorption and bioaccumulation in living biomass as well as biosorption in dead cells of different *Bacillus sphaericus* strains. The biosorption and bioaccumulation processes performed by living cells of the two most tolerant strains were similar. Biosorption in surface molecules (e.g., S-layer proteins) contributes to entrap metal ions either in living or dead cells, whereas bioaccumulation through helper proteins is involved in the incorporation of essential nutrients (e.g., P and S; Suarez and Reyes, 2002) and/or metal reduction through enzymatic processes (Elangovan et al., 2005).

#### Bioleaching

Mesophilic bacteria [such as sulfur-oxidizing bacteria (SOB; e.g., *Acidithiobacillus thiooxidans*, *A. caldus*, and *A. albertis*)] and iron-oxidizing bacteria (FeOB; e.g., *A. ferrooxidans* and *Leptospirilum ferrooxidans*; Wong et al., 2004), thermophilic bacteria (e.g., *Archeans* sp., *Sulfobacillus thermosulfidooxidans, S. ambivalens*, *S. brierleyi*, and *Thiobacter subterraneus*; Kletzin, 2006) as well as heterotrophic bacteria (e.g., *Acetobacter*, *Acidophilum*, *Arthrobacter*, and *Pseudomonas*) and fungi (e.g., *Penicillium*, *Aspergillus* and *Fusarium* and *Trichoderma*; Mulligan and Galvez-Cloutier, 2003) are able to bioleach heavy metals from sludge, sediments and soils, therefore alleviating metal phytotoxicity directly or indirectly through various metabolic activities such as oxidation, reduction, complexation, adsorption, or dissolution (Pathak et al., 2009; **Figure 3**). Kumar and Nagendran (2009) demonstrated that the acidophilic SOB *Acidithiobacillus thiooxidans* created acidic conditions favorable for bioleaching/removal of metals (e.g., Cd, Cr, Cu, Fe, Pb, and Zn). The capability of bioleaching depends on bacterial species. In general, acidophilic bacteria are more capable for metal bioleaching than neutrophilic bacteria (Navarro et al., 2013).

#### Bioexclusion

Microbial active transport or eﬄux of toxic metals from their cytoplasm represents the largest category of metal resistance systems (**Figure 3**). Non-essential metals such as Cd and As, generally enter the cell through either non-ATPase or ATPase-linked nutrient transport systems that are highly specific for the imported cation or anion (Nies and Silver, 1995), whereas active transport of essential metal ions (e.g., Cu^2+^) from bacterial cells can be achieved through an ATPase eﬄux mechanism (Bruins et al., 2000). Nies (2003) has intensively reviewed the mechanisms of eﬄux-mediated heavy metal resistance in prokaryotes by elucidating the action and physiological functions and distribution of metal-exporting proteins such as P-type ATPases, cation diffusion facilitator and chromate proteins, NreB- and CnrT-like resistance factors. Possession of the highly specialized mechanisms makes a bacterium metal resistant.

### Metal Mobilization

It is well-known that strong binding of metals to soil particles or precipitation accounts for the insolubilization of a significant fraction of metals in soil and consequently contributes to their unavailability for plant uptake. The mobility and solubilization of metals in soil have been recognized as critical factors in affecting the practical efficiency of phytoextraction (Ma et al., 2009a, 2013). In this regard, metal mobilizing microbes can be used to modify rhizosphere habitat, hence, influencing metal element speciation and mobility in soil through biogeochemical cycling processes of heavy metals, mainly including acidification, protonation and chelation (Glick, 2010; Ma et al., 2011a; Rajkumar et al., 2012; Sessitsch et al., 2013; **Figure 3**).

#### Acidification

Soil pH is a key factor affecting the content and solubility/mobility of metals in soil. The mobility of most metals decreases with increasing pH (Richards et al., 2000). Soil pH is generally influenced by activities of both plants and microorganisms, and vice versa. The hydrogen ions excreted by plant roots can displace heavy metal cations that are adsorbed on soil particles, leading to acidification of the rhizosphere. Root exudates can lower the pH of rhizosphere by one or two units over that in bulk soil (Sheoran et al., 2011), therefore enhancing soil metal mobility and plant metal bioavailability in soil solution (Alford et al., 2010; Kim et al., 2010). Chen B. et al. (2014) pointed out that the inoculation of endophytic bacterium *Sphingomonas SaMR12* regulated quantity of root exudates (organic acids) from *S. alfredii*, thus substantially improving Cd bioavailability and plant absorption facility. A recent study reported that *P. myrsinacearum* RC6b significantly increased metal uptake by *S. plumbizincicola.* This was attributed to its ability to produce organic acid and solubilize insoluble tricalcium phosphate (Ma et al., 2013).

#### Protonation

Soil microbes can also acidify their environment by exporting protons to replace heavy metal cations at sorption sites (Rajkumar et al., 2012; **Figure 3**). In order to understand, predict and optimize such processes, there have been extensive attempts to model the interactions between protons, metal ions and bacterial surfaces, as well as to characterize them using spectroscopy. Giotta et al. (2011) studied the interaction of Ni^2+^ with surface protonable groups of *Rhodobacter sphaeroides* by using attenuated total reflection Fourier transform infrared (ATR-FTIR) spectroscopy. The results revealed that carboxylate moieties on the bacterial surface play a significant role in extracellular biosorption of Ni^2+^, establishing relatively weak bonds with metal ion.

#### Chelation

Upon chelation, organic chelator compounds released from both plants and microorganisms are able to scavenge metal ions from sorption sites and heavy metal-bearing minerals, thus protecting them from resorption (Gadd, 2004). To date, the natural organic chelators are known as metal-binding compounds, organic acid anions, siderophores, biosurfactants, and metallophores (Sessitsch et al., 2013).

The metal chelation through the induction of metal-binding peptides (MTs and PCs) can eliminate the phytotoxic effect of free metal ions, allowing for metal uptake, sequestration, compartmentation, xylem loading, and transport within the plant (Cai and Ma, 2002). Phytochelatins, the heavy metal-binding peptides, are synthesized from the tripeptide glutathione and/or through an enzymatic reaction catalyzed by PCs synthase (Solanki and Dhankhar, 2011). The production of PCs is immediately induced by heavy metal exposure, which is positively correlated with metal accumulation in plant tissues (Pal and Rai, 2010; **Figure 3**). In contrast to PCs, MTs, small cysteine-rich and metal-binding proteins, play essential roles in activities of various organisms (e.g., animals, plants, eukaryotic and prokaryotic microorganisms) such as metal detoxification and homeostasis through scavenging reactive oxygen species (Leitenmaier and Küpper, 2013). Bolchi et al. (2011) pointed out that the mycorrhizal fungus *Tuber melanosporum* polypeptides such as MTs (TmelMT) and PC synthase (TmelPCS) were capable of conferring an enhanced tolerance to essential (Cu and Zn) and non-essential (Cd, As, and Hg) thiophilic metal ions in yeast. Nevertheless, it is known that MTs also occur in AMF and that genes encoding several enzymes for PCs synthesis can be activated in mycorrhizal roots, therefore enhancing photosynthetic activity in mycorrhizal plants exposed to metal stress. However, these metal chelation mechanisms cannot make a major contribution to metal tolerance strategies operating in AM symbiosis (Rivera-Becerril et al., 2005).

Fe is an important micronutrient and its concentration in soil is often below the level necessary to support robust plant and microbial life due to its low solubility, especially under metal stress. Therefore, plants surmount challenges to acquire sufficient Fe through three mechanisms, namely, Strategy I (Fe solubilization by all dicots and monocots via rhizosphere acidification); Strategy II [secretion of phytosiderophores (PSs) and uptake Fe^3+^-PS]; and Strategy III (uptake Fe^3+^-microbial siderophores by plants). Many studies have shown that PSs are able to solubilize and transport metals by chelation, and thus being secreted into the rhizosphere through a potassium-mugenic acid symporter (Sakaguchi et al., 1999). It has been proved that the siderophores produced by microorganisms have a higher affinity for metals than PSs. Hence, microbes may develop strategies to solubilize metals for a more efficient uptake by plants (**Figure 3**). Recently, Yuan et al. (2014) demonstrated that inoculation with the endophytic bacterium *Rahnella* sp. JN27 promoted Cd solubilization in metal-amended soils through the release of siderophores, therefore facilitating Cd uptake by Cd-hyperaccumulators *Amaranthus hypochondriacus* and *A. mangostanus*.

The organic acids released from both host plants and microbes have been proposed to be involved in various processes occurring in the rhizosphere, including nutrient acquisition, mineral weathering, heavy metal detoxification, and mobilization/solubilization in soil (Rajkumar et al., 2012; **Figure 3**). Organic acids excreted by plant roots, such as malate, citrate, acetate, and oxalate, are widely recognized to be responsible for dissolving the solid phase metals in soil through complexation reaction and thus making them available for plant uptake (Berkelaar and Hale, 2003; **Table 1**; **Figure 1**). Mucha et al. (2005) found that the malonate and oxalate released by *Juncus maritimus* acted as complexing agents of trace metals, and were responsible for the enhanced metal mobility and availability in soil. However, the excretion of organic acids by microorganisms has a somehow more profound effect on the stimulation of metal release than the direct effect of root secretions (Amir and Pineau, 2003). Exudates of LMWOAs from microbial populations, including both aliphatic and phenolic acids, have great potential to improve metal solubilization processes (Rajkumar et al., 2012). A recent study by Chen L. et al. (2014) demonstrated that the organic acids producing endophytic bacterium *Pseudomonas* sp. Lk9 improved soil mineral nutrition (Fe and P) and metal availability by accelerating host-mediated LMWOAs secretion, thereby significantly enhancing shoot biomass production of *Solanum nigrum* and accumulation of metals (Cd, Cu, and Zn) in aerial plant parts. Nevertheless, AMF have not been shown to produce organic acids. However, the specific protein glomalin produced by AMF seems to be efficient in sequestering heavy metals outside the mycelium (Gonzales-Chavez et al., 2004).

Biosurfactants (BSs) are amphiphilic compounds either produced on microbial cell surfaces or excreted extracellularly, and contain hydrophobic and hydrophilic moieties that reduce surface and interfacial tensions (**Figure 3**). The structures of BSs are usually composed of one or more compounds, such as mycolic acid, glycolipids, polysaccharide-lipid complex, lipoprotein or lipopeptide, phospholipid, or the microbial cell surface itself (Pacheco et al., 2011). Due to their amphiphilic structure, BSs are able to create complexes with metals at the soil interface and desorb metals from soil matrix to the soil solution, hence, increasing metal solubility and bioavailability in metal polluted soils (Sheng et al., 2008). Therefore, the use of metal resistant bacterial strains capable of producing BSs has been considered as a promising strategy to improve the removal of heavy metals from soils (Rajkumar et al., 2012; Venkatesh and Vedaraman, 2012). Recently, surfactin from *B. subtilis*, sophorolipids from *Torulopsis bombicola*, and di-rhamnolipids and rhamnolipids from *P. aeruginosa* have been employed to remove metals from contaminated soils (Mulligan et al., 2001; Juwarkar et al., 2007; Venkatesh and Vedaraman, 2012). Although, some studies have reported the potential of microbial BSs to facilitate metal mobilization, the mechanisms (such as ion exchange, precipitation-dissolution, and counterion binding) involved in the interaction among biosurfactants producing microbes, plants, and metals have been scarcely demonstrated.

Metallophores are low molecular weight organic ligands released from microorganisms, which can regulate the intracellular metal concentrations to avoid toxicity or maintain appropriate concentrations for their growth (**Figure 3**). Deicke et al. (2013) reported that metallophores (e.g., protochelin and azotochelin) excreted by the NFB *Azotobacter vinelandii* bound metal cations and oxoanions in its extracellular medium, therefore increasing the metal (Fe and Mo) bioavailability to bacterial cells, which can subsequently recruit the complexes. The efficient quantification of those metal complexes is crucial for essential cofactors of nitrogenase (e.g., Fe and Mo) homeostasis, N_2_ fixation dynamics and N_2_ cycle. This strategy allows microbes to control metal speciation in their environment, thereby increasing heavy metal bioavailability to the N_2_ fixers.

### Metal Immobilization

Some microbes can also reduce plant metal uptake or translocation to aerial plant parts by decreasing metal bioavailability in soil via precipitation, alkalinization, and complexation processes (**Figure 3**).

#### Precipitation

Certain plant associated microorganisms have the ability to promote the enzymatically catalyzed precipitation of radionuclides (e.g., U, Tc) and toxic metals (e.g., Cr, Se) by microbial reduction processes, which show considerable promise for phytoremediation of metal contaminated soils (Payne and DiChristina, 2006). Oves et al. (2013) reported that the inoculation of Cr reducing bacterium *P. aeruginosa* OSG41 onto chickpea grown in Cr^6+^ contaminated soils significantly decreased Cr uptake by 36, 38, and 40% in roots, shoots and grains, respectively, with a concomitant increase in plant growth performance compared with non-inoculated control. The results indicate that bacteria possessed the ability to protect host plant against the inhibitory effect of high concentration of Cr^6+^ by reducing mobile and toxic Cr^6+^ to non-toxic and immobile Cr^3+^ in the soil. Besides, insoluble mineral forms of radionuclides and metals can also be immobilized either directly through an enzymatic action (Pagnanelli et al., 2010), or indirectly by bacterial Fe oxidation or interactions between microbial inorganic acid (e.g., hydrogen sulfide, bicarbonate, and phosphate; Park et al., 2011; Zhou et al., 2013). The microbial inorganic acid can rapidly react with certain dissolved metals (such as Cu, Fe, Zn, and Pb) to form insoluble precipitates. Noticeably, anaerobic reduction of sulfur by sulfate-reducing bacteria (SRB) has been proved to be a promising treatment of a variety of S-containing and metal rich mining drainage and industrial eﬄuents (Zhou et al., 2013). Similarly, Park et al. (2011) reported that application of PSB reduced Pb availability in contaminated soils though the release of P from insoluble P compounds. In this sense, the ability of bacteria to solubilize minerals, promote plant growth, and immobilize metals in soil makes them a promising choice for phytostabilization of metal contaminated soils.

#### Alkalinization

Some AMF and bacteria (e.g., SRB and cyanobacteria) exhibit the ability to absorb metals through substratum alkalinization activity, therefore affecting the metal stability in soil (Büdel et al., 2004). The alkalinizing effect induced by AMF through the release of OH^-^, can result in an active nitrate uptake by microbes and a reduction in metal phytoavailability in the rhizosphere by secreting glomalin (Giasson et al., 2008). AMF can act as metal sinks to reduce the mobile and available metal cations in soil, thereby creating a more suitable environment for plants growing in metal contaminated soils (Göhre and Paszkowski, 2006). Hu et al. (2013) observed that inoculations of AMF *Glomus caledonium* and *G. mosseae* onto *S. alfredii* and *Lolium perenne* significantly decreased soil DTPA-extractable Cd by 21–38% via alkalinization process, thus facilitating both extraction and stabilization of Cd in the *in situ* treatment of Cd-contaminated acidic soil.

#### Complexation

The excretion of EPSs by plant associated microbes is of particular importance to form a protective barrier against harmful effects through metal biosorption (Slaveykova et al., 2010; Hou et al., 2013; **Figure 3**). The mechanisms involved in metal biosorption onto to EPS include metal ion exchange, complexation with negatively charged functional groups, adsorption and precipitation (Zhang et al., 2006). Guibaud et al. (2005) found that the EPS extracted from bacterial cultures were less able to complex metals than those from sludge. However, the bacterial EPS exhibited great capacity to bind metals and protect bacteria from metal stress. In addition, AMF can produce an insoluble metal-sorbing glycoprotein (glomalin) that reduces metal mobility or sequesters metals and it could be considered for metal biostabilization in soil (Vodnik et al., 2008). Wu et al. (2014) investigated the role of glomalin-related soil protein (GRSP) that is used to estimate AMF-derived glomalin in soils in sequestering Pb and Cd in an *in situ* field experiment. It was found that after 140 days GRSP bound Pb accounted for 0.21–1.78% of the total Pb, and for Cd, 0.38–0.98% of the total Cd content in the soil. Although, the metal-binding levels are insignificant compared to soil organic matter basis such as humic and fulvic acids, GRSP greatly influenced metal uptake in contaminated soils. Therefore, it is clear that AMF can reduce metal mobility in soil by excreting glomalin, however, studies concerning the structures and mechanisms of glomalin are still required to provide further knowledge.

### Metal Transformation

The activity and importance of microbes in participating in biogeochemical redox processes, which lead to diverse chemical transformation of metal contaminants, have been documented in previous studies (Chatterjee et al., 2009; Olegario et al., 2010; Majumder et al., 2013). Heavy metals, such as As, Cr, Hg, Fe, Mn and Se, are most commonly subjected to microbial oxidation and reduction reactions, thereby altering their speciation and mobility in soil and simultaneous reducing metal phytotoxicity (Kashefi and Lovley, 2000; O’Loughlin et al., 2003; Amstaetter et al., 2010; Olegario et al., 2010; **Figure 3**). In general, metals such as Cu and Hg are more soluble in their lower oxidation state, whereas the oxidation states of metals such as Cr, As, and Se are more soluble and toxic (Ross, 1994). Majumder et al. (2013) reported that the metal resistant As-oxidizing bacteria *Bacillus* sp. and *Geobacillus* sp. isolated from arsenic-contaminated soils efficiently oxidized mobile toxic As^3+^ to immobile less toxic As^5+^. In addition, Cr reduction is other example for the precipitation of metallic ions in aqueous solutions or soils. Chatterjee et al. (2009) pointed out that the inoculation of Cr-resistant bacterium *Cellulosimicrobium cellulans* decreased Cr^6+^ uptake in the shoot and root of green chili by 37 and 56%, compared with non-inoculated controls. This is due to the ability of bacteria to reduce the mobile and toxic Cr^6+^ to immobile and non-toxic Cr^3+^ in the soil. Therefore, metal reducing or oxidizing microbes are able to reduce the phytotoxic effects of metals by transforming a metal contaminant into a non-bioavailable state in the rhizosphere, reflecting the suitability of these microbes for phytotransformation.

### Metal Transport and Distribution

The translocation of metals from plant roots to the above-ground parts varies considerably depending on plant species and metals. Different metals are differently mobile within a plant, e.g., Cd and Zn are more mobile than Cu and Pb. During the transportation through the plant, metals are bound largely on the cell walls of roots, leading to high metal concentration in plant roots. The metal chelation with ligands (e.g., organic acids, amino acids, and thiols) facilitates the metal movement from roots to shoots (Zacchini et al., 2009; **Figure 3**). Due to the high cation exchange capability of the xylem cell, the metal movement is severely retarded when the metals are not chelated by ligands. Organic acids are involved in the Cd translocation in *Brassica juncea* (Salt et al., 1995), whereas, histidine is involved in the long distance Ni translocation in hyperaccumulator *Alyssum lesbiacum* (Solanki and Dhankhar, 2011). Since most heavy metals can only be transferred by forming organic compounds-metal complexes (Maser et al., 2001), a variety of organic ligands secreted by microbes can change the exiting forms and distribution of metals through combination with metals in plants, consequently facilitating the metal translocation from roots to their shoots and therefore improving phytoextraction efficiency (Sheng et al., 2008). Saravanan et al. (2007) reported that the Zn mobilizing *Gluconacetobacter diazotrophicus* helped in the efficient solubilization of the insoluble Zn compounds by producing 5-ketogluconic acid. Similarly, Ma et al. (2013) reported that application of metal resistant PGPB *P. myrsinacearum* RC6b effectively mobilized metals (Cd, Zn and Pb) in soil and significantly increased metal accumulations (Cd and Zn) in shoots of *S. plumbizincicola*. Besides, a few studies found that the growth and metal accumulation in the above ground part of plants have been improved by using combinations of plant associated microorganisms. Zimmer et al. (2009) found that inoculation of *Salix viminalis x caprea* with ectomycorrhiza associated bacteria *Micrococcus luteus* and *Sphingomonas* sp. in combination with the fungus *Hebeloma crustuliniforme* increased the total Cd and Zn accumulation in shoot up to 53 and 62%, respectively, and consequently led to an increase in phytoextraction of Cd and Zn in these fungal-bacterial inoculant combinations. Similarly, De Maria et al. (2011) observed that after inoculation with the rhizobacteria *Streptomyces* sp. and *Agromyces* sp. plus the fungus *Cadophora finlandica* onto *Salix caprea*, shoot concentrations of Cd and Zn were mostly increased, indicating higher translocation of metals from roots to shoots.

As discussed above, the functions of specific PGPM contributing to metal availability in soils have been studied at the laboratory scale. However, more attention should be given to the application of multi-functional PGPM or multi-strain inoculation exhibiting stress resistance and plant beneficial traits when used as bioinoculants with remediating plants at the field scale.

## Conclusion and Recommendations

In this review, the most important properties of plants and microbes as well as mechanisms underlying plant-microbe-metal interactions in phytoremediation were discussed through the following aspects: (1) providing deep insights on biochemical and molecular mechanisms of plant–microbe interaction, which could contribute to evolution dynamics of microbial consortia; (2) demonstrating the effectiveness of microbes devoted to hold potential stress-reducing properties and conferring their host plants metal stress resistance by acting as bioprotectants; (3) improving knowledge of how beneficial plant–microbe association may contribute to develop microbial inoculants and to promote plant yield respecting ecosystem biodiversity and safety by acting as biofertilizer; and (4) elucidating the mechanisms of cooperative plant–microbe interactions during metal detoxification, mobilization, immobilization, accumulation, translocation and transformation, which could contribute to the implementation of phytoremediation technologies.

The future research should be focused on: (1) the mechanism of plant-microbe-metal interactions under stressful environmental conditions; (2) the effectiveness of co-inoculation of PGPB and AMF response to multiple biotic and/or abiotic stress; (3) the identification of functional genes of beneficial microbes for growth enhancement and metal metabolism; (4) the optimization of techniques for application in large scale polluted fields; and (5) the exploration of commercial production of bioinoculants for use in metal decontamination.

## Author Contributions

YM developed the ideas and wrote the manuscript; RO and CZ revised the manuscript; HF was the project sponsor.

## Conflict of Interest Statement

The authors declare that the research was conducted in the absence of any commercial or financial relationships that could be construed as a potential conflict of interest.
